# Occupationally Exposed and General Population Antibody Profiles to Influenza A Viruses Circulating in Swine as Indication of Zoonotic Risk

**DOI:** 10.3201/eid3208.251995

**Published:** 2026-08

**Authors:** Celeste A. Snyder, Garrett M. Janzen, Giovana Ciacci Zanella, Daniel C.A. Moraes, Gustavo S. Silva, Jefferson J.S. Santos, Elizabeth M. Drapeau, Scott E. Hensley, Tavis K. Anderson, Phillip C. Gauger, Amy L. Baker

**Affiliations:** National Animal Disease Center, US Department of Agriculture Agricultural Research Service, Ames, Iowa, USA (C.A. Snyder, G.M. Janzen, G. Ciacci Zanella, T.K. Anderson, A.L. Baker); Iowa State University College of Veterinary Medicine, Ames (C.A. Snyder, G. Ciacci Zanella, D.C.A. Moraes, G.S. Silva, P.C. Gauger); University of Pennsylvania Perelman School of Medicine, Philadelphia, Pennsylvania, USA (J.J.S. Santos, E.M. Drapeau, S.E. Hensley)

**Keywords:** influenza, viruses, respiratory infections, zoonoses, One Health, occupational exposure, influenza A virus, hemagglutination inhibition tests, immunity, risk assessment, epidemiology, orthomyxovirus type A, porcine

## Abstract

Persons with occupational exposure to swine might be at disproportionate risk for zoonotic swine influenza A virus. To evaluate human antibody responses, we tested serum or plasma from swine veterinarian, farm employee, and general population cohorts by hemagglutination inhibition assays against representative swine and human seasonal influenza vaccine strains. We analyzed hemagglutination inhibition data by antigenic cartography to assess strain relationships and reproduction number modeling to evaluate pandemic potential using age-stratified immunity profiles. Occupationally exposed groups had lower human seasonal vaccine uptake (45.5% vs. 70%) and lower odds of seropositivity to several H1 and H3 strains from swine than did general population cohorts. One swine strain exhibited significant antigenic drift (3.62 antigenic units) from its nearest vaccine strain. Multiple strains required lower reproduction number thresholds for pandemic spread (1.09–1.35) than recorded pandemic strains (1.46–1.80), demonstrating that population immunity gaps heighten zoonotic risk to circulating swine H1 and H3 strains.

Influenza A viruses (IAVs) from the family Orthomyxoviridae cause respiratory disease in humans and swine. Four influenza pandemics have occurred since 1918, all with gene segments attributed to animal-origin IAVs. The 1918 H1N1 virus was thought to arise from a reassorted human and avian influenza virus (*1*). The 1957 H2N2 and 1968 H3N2 pandemics were also reassorted viruses containing gene segments derived from human seasonal and avian-origin IAVs (*2*). The 2009 pandemic strain (pH1N1) was a reassortment of viruses containing gene segments derived from avian, swine, and human IAVs (*3*), transmitted from pigs to humans before spreading globally (*4*,*5*). Viruses circulating in swine pose a threat to public health; IAVs in swine resulted in 578 confirmed zoonotic cases in the United States since 2010 (*6*). As a consequence, determining strains with zoonotic potential through measurements of genomic diversity, antigenic drift relative to human seasonal or candidate vaccine viruses, and human population immunity derived from vaccination or natural IAV infection is critical.

Commercial swine farms in the United States have endemic circulation of H1N1, H1N2, and H3N2 IAVs. Each subtype has substantial genomic diversity from historic human-to-swine introductions of H1, H3, N1, and N2 gene segments that established unique lineages, along with pH1N1 viruses (*7*). Three lineages of H1 viruses are frequently detected in pigs globally: classical swine (1A), which emerged coincident with the 1918 influenza pandemic; pre-2009 human-seasonal spillovers (1B); and Eurasian avian origin (1C) viruses (*8*). Human seasonal H3 viruses have been regularly introduced into swine populations since the 1968 H3N2 pandemic; lineages are grouped by decade of introduction (*9*). Because swine are susceptible to both avian and human IAVs, in addition to endemic swine-adapted strains, reassortment might result in novel genotypes with increased zoonotic potential (*10*,*11*).

The evolution of IAVs in swine results in antigenically drifted viruses, to which humans might have little prior immunity (*12*,*13*). Immune profiles to IAVs derived from vaccination or exposure to IAVs are not homogenous. Numerous demographic factors, such as age, sex, ethnicity, income, and health insurance status, influence influenza vaccination rates among adults in the United States, subsequently affecting their potential protection against influenza (*14*). Veterinarians are at increased risk for infection with many zoonotic pathogens (*15*). In addition, farm employees working at the human–swine interface might be at heightened risk for zoonoses (*16*–*18*).

Understanding human population immunity to endemic IAV in swine, particularly among occupationally exposed persons with regular swine contact, provides critical context for zoonotic risk assessments. This study quantified antibody response within different human populations to representative strains of IAV in US swine to evaluate whether occupation or other demographic factors increased zoonotic risk. We tested swine antigens from the most frequently detected IAV hemagglutinin (HA) clades in US swine against human serum samples derived from 2 occupationally exposed cohorts collected in the United States, swine veterinarians and swine farm employees, and 2 general population cohorts from the United States and Hong Kong, China. Profiling human antibody titers against circulating IAVs in swine might highlight immunologic gaps across populations to enable early identification of high-risk strains before zoonotic outbreaks.

## Materials and Methods

### Human Cohorts

We collected human serum or plasma samples to assess antibody cross-reactivity to IAVs in swine. Occupational cohorts included veterinarians from a swine conference in the fall of 2021 (self-identified as swine-focused) and employees from 5 Midwest US swine farms (2022). Occupational cohorts completed a questionnaire on demographics, swine contact history, and recent seasonal influenza vaccination. General population cohorts included participants from a vaccination study at the University of Pennsylvania, sampled 28 days postvaccination (2021). For the purpose of comparison to a separate geographic region without circulation of the US swine IAV, the fourth cohort consisted of serum samples from an observational population immunity study at the University of Hong Kong, China (2022). Data on age or decade of birth, sex, and vaccination status were provided for both general population cohorts. The 4 cohorts were labeled Veterinarian, Farm Employee, Philadelphia, and Hong Kong. 

Written informed consent was obtained from all participants. The study protocols were approved by the institutional review boards of the University of Hong Kong (approval no. UW 19-720), University of Pennsylvania (approval no. 849398), and Iowa State University (approval no. 21-184).

### Representative IAVs in Swine and Reference Human Seasonal Virus Selection

We selected IAV strains to represent the genetic diversity of viruses circulating during the period of human sample collection. We queried the octoFLUdb database (*19*) for swine HA genes collected during July 2021–June 2022. The data included 686 H3 HA genes and 1,294 H1 HA genes from 25 US states, covering 98.2% of the US swine population (*20*). After alignment with mafft version 7.526 (*21*), we inferred maximum-likelihood trees using IQ-Tree version 2.2.2 (*22*) with automatic model selection (*23*). For each HA clade, we made a representative selection using PARNAS version 0.1.6 (*24*) and requested isolates from the US Department of Agriculture–administered surveillance system of IAV in swine (*19*), including 7 H1 viruses from the 1A classical swine and 1B human-seasonal lineages and 3 H3 viruses from 3 different lineages (Table). The 1A viruses share a common ancestor and emerged in pigs coincident with the 1918 influenza pandemic (*1*). The 1A.3.3.2 (pH1N1) and 1A.3.3.3 HA genes share a common ancestor in the early 2000s (*5*). The 1B.2.1 and 1B.2.2.x viruses in the United States resulted from 2 independent human-to-swine spillovers of pre-2009 human seasonal viruses in ≈2003 (*2*). The H3 viruses share a common ancestry with human seasonal H3: from the mid-1990s for the 1990.4 lineage, ≈2012 for the 2010.1 lineage, and ≈2016 for the 2010.2 lineages. We downsampled the inferred trees for visualization using smot version 0.17.4 (*25*). The virus antigen panel included contemporary human seasonal H1 and H3 vaccine strains (HuVac) derived from the World Health Organization–recommended 2020–2021 Northern Hemisphere influenza vaccine strains and a pre-2009 H1 vaccine strain that shared a common ancestor with the swine H1 1B strains. (Extended methods and code are available at https://github.com/flu-crew/datasets.)

**Table:** Representative influenza A virus antigens from swine and human seasonal influenza vaccines included in hemagglutination inhibition assays in study of occupationally exposed and general population antibody profiles to influenza A viruses circulating in swine as indication of zoonotic risk*

Strain	Abbreviation	Subtype	HA clade†	NA clade
rg-A/Hawaii/70/2019	HI19.HuVac	H1N1	6B.1A.5a.1	B.2
A/swine/Illinois/A02524514/2020	IL20.1A.1.1.3	H1N2	1A.1.1.3	N2–1998B
A/swine/Iowa/A02524480/2020	IA20.1A.3.3.2	H1N1	1A.3.3.2	N1-pdm
A/swine/Indiana/A02636365/2022	IN22.1A.3.3.3-c1	H1N1	1A.3.3.3-c1	N1-classical
A/swine/Minnesota/A02635908/202	MN21.1A.3.3.3-c3	H1N1	1A.3.3.3-c3	N1-classical
A/Beijing/262/1995	BJ95.HuVac	H1N1	NA	NA
A/swine/Iowa/A02635863/2021	IA21.1B.2.1	H1N2	1B.2.1	N2–1998B
A/swine/Iowa/A02478968/2020	IA20.1B.2.2.1	H1N2	1B.2.2.1	N2–2002B
A/swine/Iowa/A02524534/2020	IA20.1B.2.2.2	H1N2	1B.2.2.2	N2-LAIV-98
A/Hong Kong /45/2019	HK19.HuVac	H3N2	3C.2a1b.1b	A.2.2
A/swine/Iowa/A02635890/2021	IA21.1990.4.a	H3N2	1990.4.a	N2–2002B
A/swine/Kansas/A02245675/2020	KS20.2010.1	H3N2	2010.1	N2–2002A
A/swine/Indiana/A02635878/2021	IN21.2010.2	H3N2	2010.2	N2–2016

*HA, hemagglutinin; NA, not applicable. †HA lineage and clade designations: 1A = classical swine (1918 spillover), 1B = human seasonal spillover (2000s), 1990.4 and 2010.x = human H3N2 spillovers (1990 or 2010 decade indicated).

### Serology

We treated serum and plasma samples with receptor-destroying enzyme (RDE; Sigma-Aldrich, https://www.sigmaaldrich.com) at 37°C for 18–20 hours. We inactivated RDE at 56°C for 30 minutes and absorbed serum samples using packed turkey red blood cells at 0.5% for 1 hour to remove nonspecific agglutinins. We expanded representative swine strains and human seasonal influenza vaccines in Madin-Darby canine kidney cells and tested them against human serum and plasma in hemagglutination inhibition (HI) assays (*26*). We calculated geometric mean titers (GMT) by the log2 transformation of reciprocal titers divided by 10. HI titers of <10 were given a pseudo count 10 to permit log2 transformation. We considered a reciprocal HI titer of >40 or a log2 scale of 2 positive and a correlate of protection. That threshold is for seasonal human influenza characterization; although it has not been validated for swine-to-human cross-protection, we maintained this metric for our zoonotic risk assessment.

### Antigenic Characterization

We generated antigenic maps with HI assay data using Racmacs (*27*) (https://acorg.github.io/Racmacs) in R version 4.4.1 (The R Project for Statistical Computing, https://www.r-project.org). We used Euclidean distances between pairs of antigens to determine the antigenic distances between selected IAVs circulating in swine and human seasonal vaccine strains. We calculated antigen-serum distances to determine the proportion of persons with a significant antigenic distance from circulating swine strains. One antigenic unit (AU) equates to a 2-fold difference in HI titer. We used an antigenic distance of >3 AU (8-fold difference in HI titer) as a threshold to indicate a potential need to revise human seasonal influenza vaccine components (*28*).

### Odds Ratio Analysis

We calculated odds ratios (ORs) using cohort type (occupational exposure and general population), birth year (divided into groups of 1946–1977 and 1978–2003), sex, and vaccination status to evaluate how those factors influenced the odds of seropositivity (reciprocal HI titer >40). Birth decade was not included because of small sample sizes within individual decades, although we included descriptive analysis of seropositivity by birth decade (Appendix Figure 1). The birth year cutoff of 1977 represents the reemergence of H1N1 after the H2N2 and H3N2 pandemics. We calculated OR analyses in R version 4.4.1 with the epitools package (https://cran.r-project.org/web/packages/epitools), using median-unbiased estimation and Fisher exact methods for CI calculation. We did not calculate CIs for contingency tables containing >1 values of zero. We conducted OR analyses for composite groups of occupational exposure (veterinarian + farm employee) and general population (Philadelphia + Hong Kong) to increase sample size and improve precision.

### Modeling Reproduction Number Thresholds from Serologic Data

We calculated reproduction number (R_0_) threshold estimates using age-stratified HI data from combined cohorts into 7 age groups (0–19, 20–29, 30–39, 40–49, 50–59, 60–69, >70 years). This approach models the theoretical minimum viral transmissibility requirements for pandemic emergence on the basis of population immunity gaps, rather than measuring basic R_0_ values from transmission data. Reciprocal titer values ranged from <10 to 10,240; values <10 were assigned a value of 5. Parameters were derived from previously described studies (*29*). We estimated values with 500 Monte Carlo bootstrap simulations, incorporating a previously described empirical contact matrix, aggregated to align with age stratification (*30*) and adaptive titer-protection relationships. Protection levels ranged linearly from 0% to 95% across titer levels, following prior pandemic risk assessment approaches (*31*). That approach estimated the population immunity proportion, relative R_0_ reduction, GMT, and minimum R_0_ threshold required for pandemic emergence. The R_0_ threshold reflects the minimum inherent viral transmissibility needed to overcome existing population immunity and achieve sustained human-to-human transmission.

## Results

### Human Cohorts Demographics

The veterinarian cohort (n = 51) consisted of 38 (74.5%) men and 13 (25.5%) women; the median age was 43 years. In the self-reported questionnaire, 31 (60.8%) reported recent seasonal influenza vaccination. The cohort reported residences across Iowa, Kentucky, Illinois, Indiana, Nebraska, Missouri, Minnesota, North Carolina, Pennsylvania, and Kansas. The farm cohort (n = 47) came from farms in Iowa, Illinois, and South Dakota; 29 participants (61.7%) were men and 18 (38.3%) were women. Median age was 37 years. Among farm employees, 14 (29.8%) reported that they received recent seasonal influenza vaccination. Persons in the farm and veterinarian cohorts were considered to have occupational exposure; 72% (n = 98) reported their last contact with swine within 1 day.

The Philadelphia cohort (n = 40) consisted of 17 (42.5%) men and 23 (57.5%) women; median age was 33 years. All (100%) persons received the 2021–2022 human seasonal influenza vaccine. The Hong Kong cohort (n = 48) consisted of 16 men (33.3%) and 32 women (66.7%); median age was 46 years. Among those persons, 16 (33.3%) reported receiving vaccination the prior year.

### Genetic Diversity of H1 and H3 IAV in Swine

**Figure 1:**
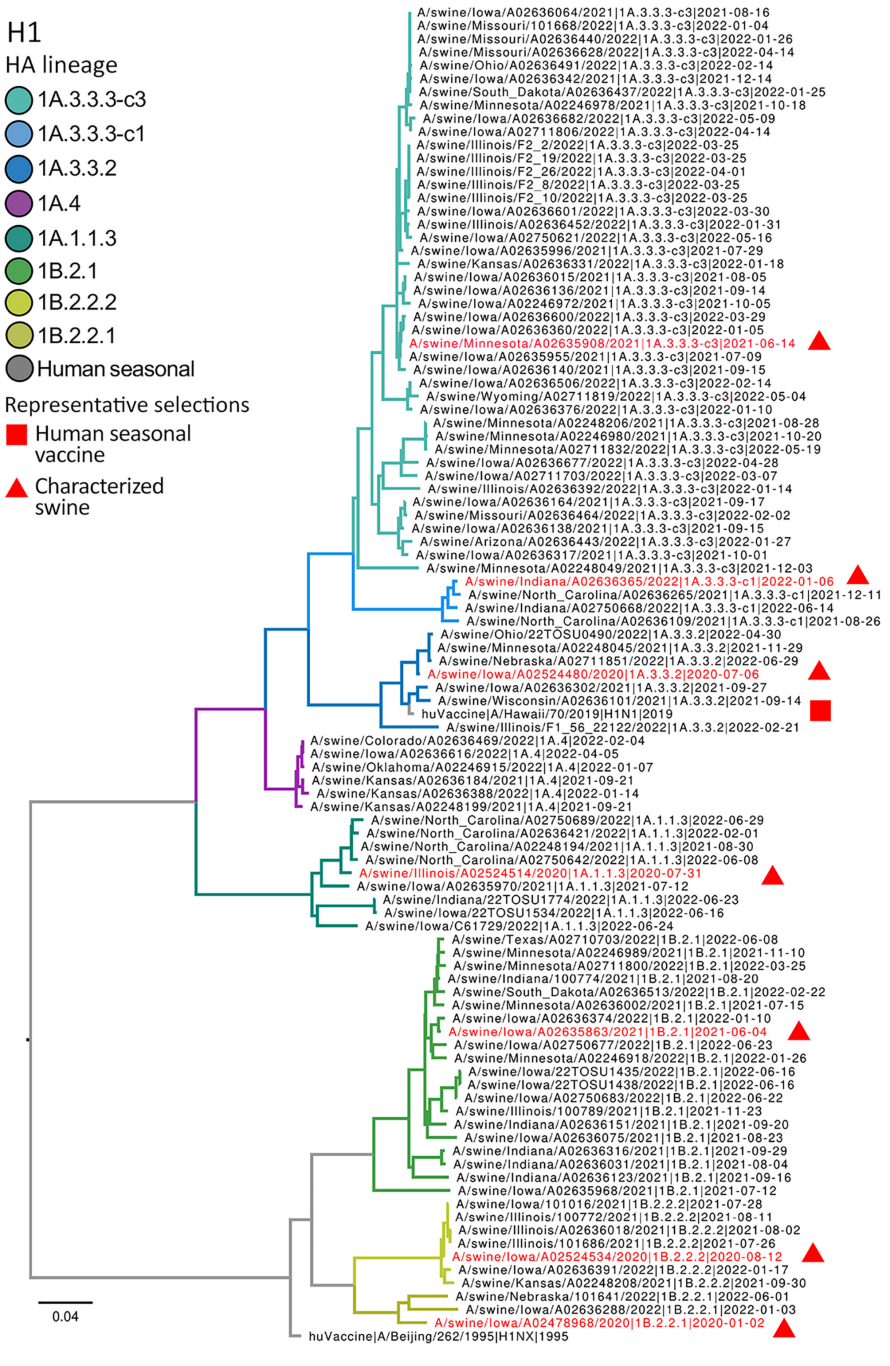
Maximum-likelihood phylogeny of swine and human influenza H1 HA genes from study of occupationally exposed and general population antibody profiles to influenza A viruses circulating in swine as indication of zoonotic risk. Two major H1 HA lineages in US swine resulted from independent introductions of H1N1 and H1N2 viruses from humans to swine and are grouped by the 1A and 1B lineage designations. Each lineage is divided and colored by statistically supported clades: 1A.1.1.3, 1A.3.3.2, 1A.3.3.3-c1, 1A.3.3.3-c3, 1A.4; and 1B.2.1, 1B.2.2.1, and 1B.2.2.2. Triangles and squares annotate selected reference and test antigens. HA, hemagglutinin.

**Figure 2:**
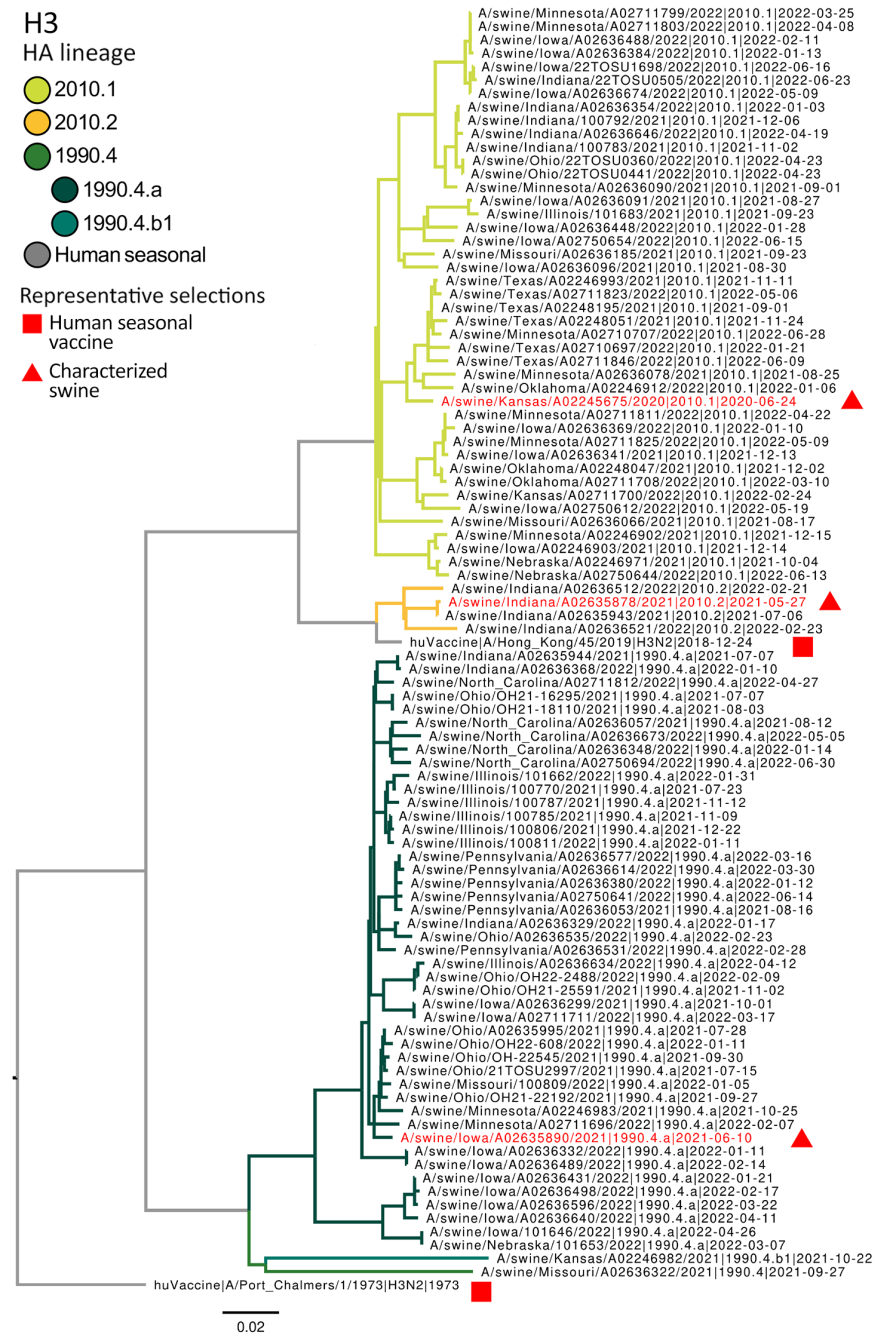
Maximum-likelihood phylogeny of swine and human influenza H3 HA genes from study of occupationally exposed and general population antibody profiles to influenza A viruses circulating in swine as indication of zoonotic risk. Three major H3 HA lineages in US swine were derived from interspecies transmission in the 1990s (1990.4.x) and 2010s (2010.1 and 2010.2). Each lineage is divided and colored by statistically supported clades. The H3 tree was rooted on the human seasonal HA gene A/Port Chalmers/1/1973 (H3N2). Branch lengths were drawn to scale. Scale bar indicates number of nucleotide substitutions per site. The phylogeny with tip labels included is available at https://github.com/flu-crew/datasets. HA, hemagglutinin.

During the human blood collection period in July 2021–June 2022, 8 H1 1A, 3 H1 1B, and 6 H3 clades from 3 lineages (1990.4, 2010.1, 2010.2) were detected from 25 US states. The H1 subtype (645 sequences, 65.4%) had more detections than the H3 subtype (341 sequences, 34.6%). The 1A.1.1.3 (10.6%), 1A.3.3.2 (7.1%), 1A.3.3.3-c3 (24.7%), and 1B.2.1 (15.7%) were the most frequently detected clades (Appendix Figure 2) (*19*). The H3 1990.4 lineage diversified to 4 distinct groups, but only the 1990.4.a (13.6%) represented >1% of detections. The H3 lineages 2010.1 and 2010.2 represented 14.5% and 1.1% of the detections. We selected 10 viruses with HA genes (Table; Figures 1, 2) representing 97.8% of detections during July 2021–June 2022 (*19*).

### Antibody Levels to IAVs in Swine by Cohort

**Figure 3:**
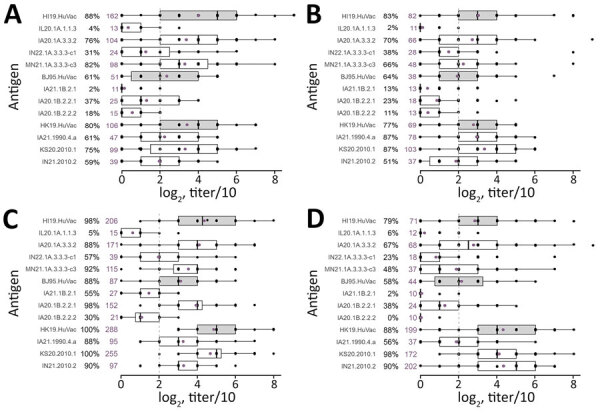
Box and whisker plots of log2 transformed hemagglutination inhibition (HI) titers among participants in 4 human cohorts against contemporary swine H1 and H3 influenza A virus strains in study of occupationally exposed and general population antibody profiles to influenza A viruses circulating in swine as indication of zoonotic risk. A) Veterinarians (n = 51); B) farm employees (n = 47); C) Philadelphia general population cohort (n = 40); D) Hong Kong general population cohort (n = 40). Box plots show median log2 transformed HI titers on the x-axis, test strains on the y-axis, interquartile ranges, and outliers. Purple dots represent the geometric mean log2 transformed HI titers/10. Gray box plots represent responses against human seasonal influenza vaccine strains, and white box plots represent responses against swine strains. Reverse-transformed HI geometric mean titers in purple and percentages of positive persons in black are shown next to virus names on each panel. The gray dotted line indicates the HI titer threshold of 40 (log2 scale of 2).

HI antibody titers to representative swine and human seasonal vaccine strains demonstrated low responses to several swine IAV strains across cohorts. Titers to IA20.1A.3.3.2 and MN21.1A.3.3.3-c-3 were higher than titers to IL20.1A.1.1.3, IN22.1A.3.3.3-c-1, IA21.1B.2.1, and IA20.1B.2.2.2 (Figure 3). Swine H3N2 representatives elicited higher GMT and percentages of seropositive persons overall, although 10.2%–28.5% of participants remained below the positive cutoff (Log2[titer/10] or 40 GMT).  In the veterinarian cohort, 88% were HI positive (GMT = 162) for the human H1N1 vaccine component, HI19.HuVac, 61% (GMT = 51) tested positive for the pre-2009 historical H1N1 vaccine strain BJ95.HuVac, and 80% (GMT = 105) tested positive for the human H3N2 vaccine component, HK19.HuVac (Figure 3, panel A). Farm cohort seropositivity was 83% (GMT = 82) for HI19.HuVac, 64% (GMT = 38) for BJ95.HuVac, and 77% (GMT = 68) for HK19.HuVac (Figure 3, panel B).

The Philadelphia cohort displayed the highest responses with 98% (GMT = 206) HI positivity for HI19.HuVac, 88% (GMT = 87) for BJ95.HuVac, and 100% (GMT = 288) for HK19.HuVac (Figure 3, panel C). HI results for contemporary swine strains clades 1A.1.1.3, 1A.3.3.3c-1, 1B.2.1, and 1B.2.2.2 showed broader antibody cross-reactivity than in other cohorts, although some persons in every cohort were seronegative (Figure 3, panel C). Hong Kong cohort seropositivity was 79% (GMT = 71) for HI19.HuVac, 58% (GMT = 44) for BJ95.HuVac, and 88% (GMT = 199) for HK19.HuVac (Figure 3, panel D). This cohort followed similar trends to the other cohorts, except titers to IN21.2010.2, which displayed higher GMTs and percent positives like the human seasonal vaccine strain HK19.HuVac (Figure 3, panel D).

### Impact of Demographics and Vaccination on Seropositivity Patterns

**Figure 4:**
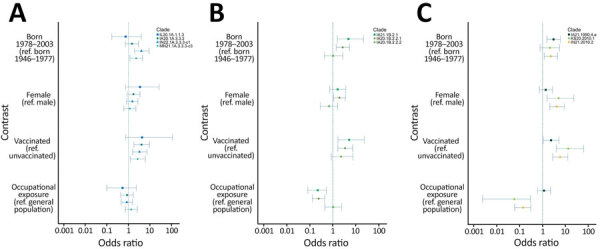
Seropositivity for influenza A virus in swine clades in study of occupationally exposed and general population antibody profiles to influenza A viruses circulating in swine as indication of zoonotic risk. Swine clades shown are H1 1A (A), H1 1B (B), and H3 (C) for demographic and vaccination status variables. Symbols indicate odds ratios; error bars indicate 95% CIs. Odds ratios displayed on logarithmic x-axis scale. Ref., referent.

Demographic factors significantly affected patterns of seropositivity across all IAV strains tested (Figure 4). Occupational exposure to swine was associated with significantly lower odds of seropositivity for H1 1B (OR 0.23 [95% CI 0.11–0.48]; p<0.001) and H3 lineages (OR 0.17 [95% CI 0.05–0.44]; p<0.001) compared with general population cohorts. Uptake of the human seasonal vaccine produced the strongest seropositivity association across all subtypes, H1 1A (OR 5.30; p<0.001), H1 1B (OR 5.48; p<0.001), and H3 (OR 14.44; p<0.001). In addition, persons born during 1978–2003 showed higher seropositivity than those born during 1946–1977, for H1 1A (OR 1.94; p = 0.038), H1 1B (OR 3.47; p = 0.002), and H3 (OR 2.35; p = 0.036) lineages (Appendix Table 1).

At the strain level, tested H1 1B strains showed vulnerabilities in the occupational cohorts across the lineage (OR 0.23); IA21.1B.2.1 and IA20.1B.2.2.1 demonstrated strong negative associations (ORs 0.22–0.24; p<0.001) (Appendix Table 2). H3 lineages displayed the strongest overall negative association with occupational exposure (OR 0.16), although that result was driven primarily by KS20.2010.1 (OR 0.06; p<0.001) (Appendix Table 3).

### Antigenic Distance between IAVs in Swine and Representative Human Seasonal Vaccine Strains and Seropositive Individuals

**Figure 5:**
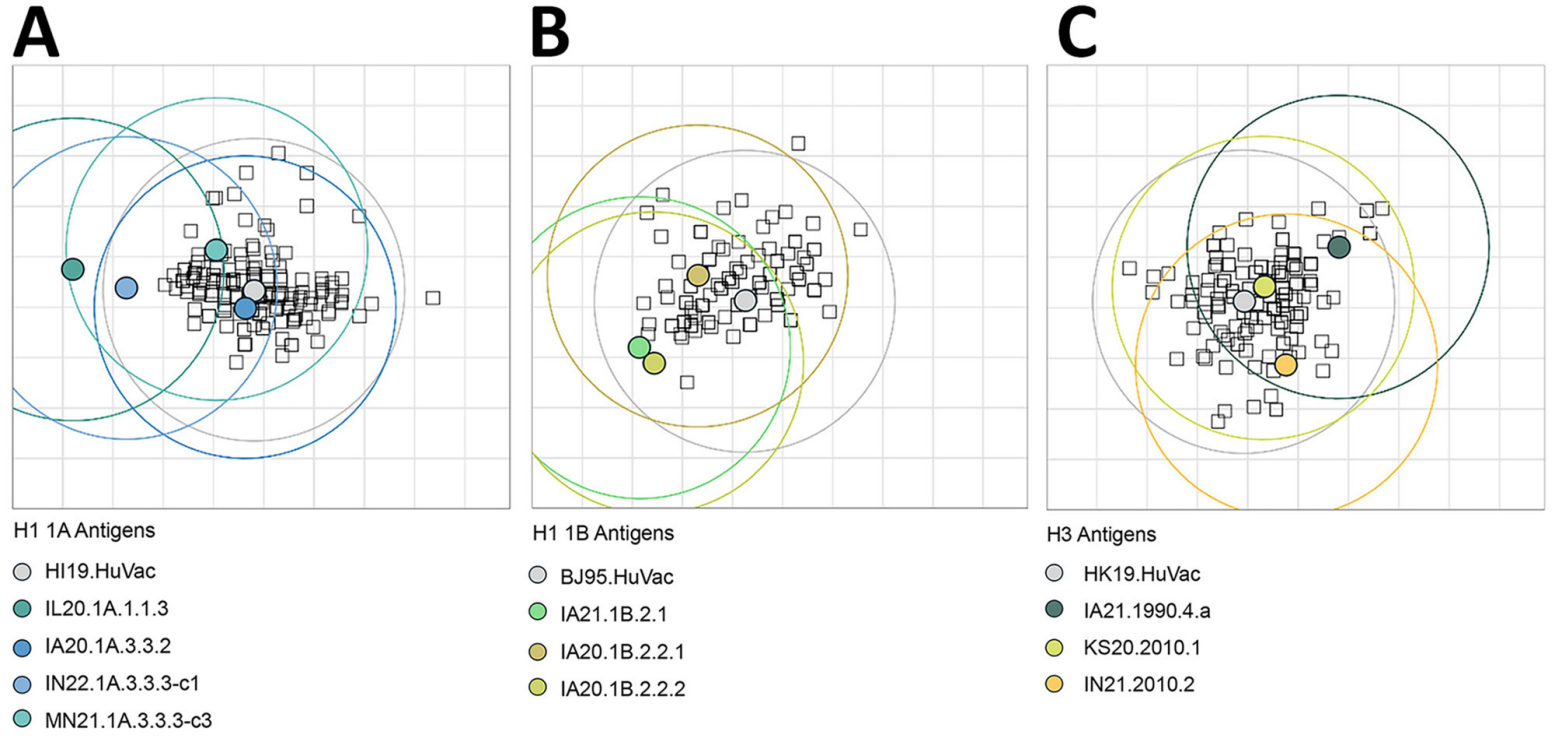
Antigenic map showing the relationships between human antibody immunity and selected strains of influenza A virus (IAV) in swine and human seasonal vaccines in study of occupationally exposed and general population antibody profiles to IAVs circulating in swine as indication of zoonotic risk. Each sphere on the map represents an antigen; squares represent individual participant serum samples from the combined veterinarian, farm, Philadelphia, and Hong Kong cohorts. A) H1 1A classical swine lineage derived from the 1918 human influenza pandemic; B) H1 1B human seasonal lineage derived from introductions of pre-2009 human seasonal H1 IAV; C) H3 lineages derived from decadal introductions of human seasonal H3N2 viruses into swine. Rings around each strain display 3 antigenic units or 8-fold change in titer from the associated antigen. Persons outside the ring are predicted to have significant loss in cross-protection.

We used antigenic maps to visualize HI data, independent of vaccination status or other demographic factors, relative to the nearest human seasonal vaccine strain and displayed for each phylogenetic lineage of the swine strains (Figure 5). In the H1 1A lineage, the swine representative of the pH1N1 pandemic group (1A.3.3.2) was the most closely related antigen to the human vaccine strain HI19.HuVac (0.39AU). The 1A.1.1.3 strain displayed the highest antigenic distance from HI19.HuVac (3.62 AU); 69% of persons were >3 AU distance to the vaccine (Figure 5, panel A). However, the association between previous vaccination and responses to this strain could not be assessed because of low seropositivity (Appendix Table 4). Clade 1A.3.3.3-c1 had 31% of persons with >3 AU antigenic distance and was 2.54 AU to the vaccine strain (Figure 5, panel A). In the H1 1B lineage, the 1B.2.2.1 strain was the most closely related antigen to the historic human seasonal vaccine strain BJ95.HuVac (1.07 AU). For the clade 1B.2.1 strain, 22% of persons exhibited significant antigenic distance from the swine strain; for the clade 1B.2.2.2 strain, that percentage was 19% (Figure 5, panel B). H3 lineage antigens displayed relatively minimal antigenic distance compared with the human seasonal vaccine strain HK19.HuVac. However, 10% of persons exhibited significant antigenic distance to the 1990.4.a clade antigen (2.17 AU distance to the vaccine strain) (Figure 5, panel C).

### Assessment of Pandemic Potential from Population Immunity

**Figure 6:**
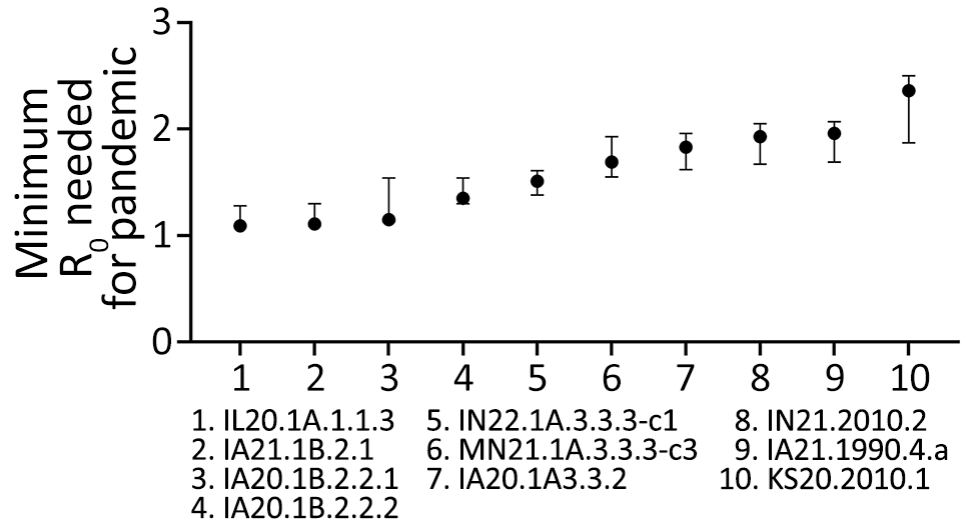
Minimum R_0_ estimates required for swine strains to cause a human pandemic derived from serologic data in study of occupationally exposed and general population antibody profiles to influenza A viruses circulating in swine as indication of zoonotic risk. Error bars represent 95% CIs. Strains are ordered by increasing minimum R_0_ thresholds. R_0_, reproduction number.

Because occupational and general population categorization was not a significant indicator of HI seropositivity, individual data for participants were combined to give better distribution across the age classes. R_0_ modeling revealed that pandemic vulnerability on the basis of human population immunity ranged from 16.3% to 52.5% across tested antigens; minimum R_0_ thresholds required for pandemic emergence ranged from 1.09 to 2.36 among the swine strains (Figure 6). Four swine strains demonstrated low R_0_ pandemic thresholds, indicating the potential to achieve pandemic spread by overcoming population immunity, with lower minimum inherent human transmissibility than the 2009 pH1N1 virus (minimum R_0_ <1.46) (*32*). The H1 1A strains had low to moderate R_0_ thresholds (range 1.09–1.83). All H1 1B lineage strains showed low thresholds (range 1.11–1.35), whereas H3 strains had relatively higher R_0_ threshold values (range 1.93–2.36). The lowest R_0_ thresholds were observed for IL20.1A.1.1.3 (minimum R_0_ = 1.09 [95% CI 1.07–1.28]) and IA21.1B.2.1 (minimum R_0_ = 1.11 [95% CI 1.09–1.30]); only 16% of persons demonstrated immunity (Appendix Figure 3), indicating those swine strains have the potential to emerge in the human population even with relatively low inherent transmissibility requirements.

## Discussion

This study assessed zoonotic risk across 4 human cohorts using population immunity and antigenic relatedness, elements of the Centers for Disease Control and Prevention’s Influenza Risk Assessment Tool (*33*). Neutralizing antibodies were measured by HI assays against a panel of representative US swine IAV and human seasonal vaccine strains. Regardless of exposure to swine, all cohorts had gaps in population immunity against several strains of IAVs circulating in US swine, indicating zoonotic transmission vulnerability. In contrast to the hypothesis that occupationally exposed persons are more likely to be infected with swine IAV and seropositive to swine strains, our findings revealed significantly lower odds of seropositivity to H1 1B and H3 swine strains for those persons compared with the general population.

Demographic variables and vaccination status significantly influenced seropositivity to swine IAVs. Whereas combining cohorts improved statistical power, it might have masked key differences in immunity patterns across groups. Occupational exposure to swine was associated with reduced seropositivity compared with general population cohorts for H1 1B (OR 0.23; p<0.001) and H3 lineages (OR 0.17; p<0.001), likely reflecting lower seasonal influenza vaccination rates. The Philadelphia cohort displayed the highest overall antibody responses because those samples were collected within a vaccine study. Although the Hong Kong cohort vaccination rate was similar to that of farm workers and showed patterns similar to both occupational groups, we noted exceptions to some strains, suggesting factors beyond vaccination contribute to those differences. Significant demographic associations included birth year; persons born after 1977 demonstrated higher seropositivity to H1 1A (OR 1.94; p = 0.038), H1 1B (OR 3.47; p = 0.002), and H3 (OR 2.35; p = 0.036). This 1978–2003 birth year effect might reflect the timing of human-to-swine spillover events when initial influenza exposures to common ancestral strains created lasting immunological imprints that resulted in cross-reactivity to related contemporary swine strains (*34*). Swine farm employees displayed low vaccination rates (33.3%), 18% less than the national coverage estimates for the 2021–2022 season (51.4%) (*35*). Vaccination was associated with correlates of protection for H1 1A (OR 5.30; p<0.001), H1 1B (OR 5.48; p<0.001), and H3 (OR 14.44; p<0.001), where seropositivity was significantly higher for vaccinated persons than for unvaccinated persons, regardless of occupational exposure. Strain-specific analyses revealed vulnerabilities; occupational groups demonstrated significantly lower seropositivity to H1 strains IA21.1B.2.1 and IA20.1B.2.2.1 (OR 0.22–0.24; p<0.001) and H3 strain KS20.2010.1 (OR 0.06; p<0.001). Persons from all cohorts lacked protective immunity against IL20.1A.1.1.3, IN22.1A.3.3.3-c-1, IA21.1B.2.1, and IA20.1B.2.2.2, indicating increased zoonotic risk for these clades of endemic swine IAV strains.

Gaps in seropositivity were further supported by the magnitude by which the swine strains differed antigenically from human vaccine strains. Whereas antibody titers from many occupational and general population persons were within an 8-fold range of representative human seasonal vaccine strains, the representative clade 1A.1.1.3 had the highest significant antigenic distance from the H1 human seasonal vaccine strain (3.62 AU), suggesting a mismatch between circulating swine viruses and existing human immunity. This result signals a need for regular assessment and update of pandemic preparedness vaccines, because the human seasonal vaccine likely provides little cross-protection against this swine H1 clade.

Four strains representing those circulating in the US swine population demonstrated minimum R_0_ thresholds <1.46, the R_0_ of the 2009 IAV pandemic from swine. Two swine strains, IL20.1A.1.1.3 (R_0_ threshold 1.09) and IA21.1B.2.1 (R_0_ threshold 1.11), were estimated to achieve pandemic spread with lower inherent transmissibility than any recorded influenza pandemic (*32*). Those 2 H1 clades were detected consistently (*19*) and represented 26.3% of the swine IAV detections across multiple US states during the study period. If the swine strains acquired moderate transmissibility in humans, existing population immunity would not be sufficient, and pandemic spread could be established.

Although the persons in this study were sampled from specific participating groups and not a random population sample, patterns of vulnerability were identified. Persons occupationally exposed to swine were significantly less likely to have protective antibody levels against contemporary IAVs in swine. Some persons might have accumulated antibodies against swine virus strains over decades, but our representative selection represents HA clades consistently detected in swine surveillance. In addition, lower seropositivity in occupational groups suggests that possible historical exposures did not provide antibody recognition against currently circulating antigens, although longitudinal studies are needed to reveal changes in antibody profiles over time. Farm workers exhibited lower vaccination rates and reduced seropositivity, underscoring a heightened risk for zoonotic infection. Those findings demonstrate that immunity profiling can identify high-risk populations and potential pandemic threats before widespread transmission occurs. Risk analysis must be paired with preventive measures to reduce IAV infections and protect public health. Swine-exposed populations, such as farm workers and veterinarians, should be targeted for seasonal IAV vaccination to reduce zoonotic risk and reduce human-to-swine transmission (*36*). Swine strains representing HA clades 1A.1.1.3, 1A.3.3.3-c-1, 1B.2.1, and 1B.2.2.2 demonstrated increased pandemic risk through low population immunity, limited cross-reactivity to seasonal vaccine strains, and low transmissibility thresholds. Candidate vaccine virus selections should prioritize those strains for regularly updated pandemic preparedness vaccine development.

This article was preprinted at https://doi.org/10.64898/2026.01.08.26343691.

Appendix Additional information about occupationally exposed and general population antibody profiles to influenza A viruses circulating in swine as indication of zoonotic risk.
